# High formin binding protein 17 (FBP17) expression indicates poor differentiation and invasiveness of ductal carcinomas

**DOI:** 10.1038/s41598-020-68454-9

**Published:** 2020-07-14

**Authors:** Prabhat Suman, Sarthak Mishra, Harish Chander

**Affiliations:** 1 Laboratory of Molecular Medicine, Department of Human Genetics and Molecular Medicine, Central University of Punjab, Bathinda, 151001 India; 2 Department of Vascular Cell Biology, Max Planck Institute for Molecular Biomedicine, 48149 Münster, Germany; 3 Immuno and Molecular Diagnostic Laboratory, National Institute of Biologicals, Noida, 201309 India

**Keywords:** Cancer, Biomarkers

## Abstract

Formin binding protein 17 (FBP17) belongs to Cdc-42 interacting protein 4 subfamily of F-BAR proteins. Recently, we had reported that FBP17 was overexpressed in invasive breast cancer cells and interacts with the actin regulatory proteins. We also reported that FBP17 promotes invadopodia formation and enhances extracellular matrix degradation. The current study determines FBP17 expression in invasive ductal carcinomas (IDCs) using breast cancer tissue microarrays (TMAs) (82 IDCs with variable receptor status and 8 Normal adjacent tissues) and its correlation with the clinico-pathological features. Immunohistochemistry of human breast cancer TMAs showed the significant elevation in the levels of FBP17 in breast cancer tissues than the normal (*p* ≤ 0.0001). Interestingly, FBP17 had a higher expression in invasive molecular subtypes HER2 and TNBC (*p* ≤ 0.05). Similarly, tumors with lymph node positive status showed elevated FBP17 expression in HER2 and TNBC subtypes (*p* ≤ 0.05). Surprisingly, grade 3 tumors demonstrated higher FBP17 expression (*p* ≤ 0.01) indicating its role in poorly differentiated tumors. Together, the data demonstrates the overexpression of FBP17 in invasive and poorly differentiated tumors. Understanding the role of FBP17 in poor differentiation and invasion of tumors in molecular subtypes at various level might represent as a potential molecular target against the disease.

## Introduction

Invasive ductal carcinomas (IDCs) are heterogeneous and categorized into various molecular subtypes that differ in the risk of invasion and metastasis^1,2^. Invasive phenotypes of breast cancer correlates with the change in cell shape and failure in the actin cytoskeleton organization leading to the formation of numerous metastatic supporting specialized structures like invadopodia, etc.,^3-6^. Fer/cip-bin amphiphysin RVS (F-BAR) proteins play a major role in the process of changes in cytoskeleton and invadopodia formation. Members of CIP4 family particularly Transducer of cdc-42 dependent actin assembly protein 1 (TOCA-1), Cdc-42 interacting protein 4 (CIP4), Formin Binding protein 17 (FBP17) have been studied previously and were found to contribute in promoting invadopodia formation and tumor metastasis^4,5,7,8^. Previously we and others described the function of CIP4 family members in invadopodia formation and showed them as an important component of invadopodia^5^. Subsequently, we also showed the expression of Toca-1 in breast cancer tissues and demonstrated its high expression in invasive breast cancer subtype^5,8^. FBP17 along with CIP4 and TOCA-1 regulate endocytosis by inducing F-actin polymerization thus promoting vesicle motility and membrane scission. These members were found to potently activate the actin polymerization^9-14^. Toca-1 and CIP4 have been shown to be overexpressed in breast cancers and play a significant role in the invasion of cancer cells,^3,5-8,14^. FBP17 contributes in the formation of podosomes and phagocytic cups required for the migration of macrophages, by recruiting WASP, WASP-interacting protein (WIP), and dynamin-2^15^. Studies regarding role of FBP17 in the process of invasion are limited. Previously, FBP17 have been shown to play a critical role in the invasion of bladder cancer cells^16^ and we have described FBP17 as an component of invadopodia in breast cancer cells^4,16^.

Regarding the role of FBP17 in breast cancer invasion, previously our findings showed a significant higher expression of FBP17 in invasive breast cancer cells. We also demonstrated that FBP17 interacts with actin regulatory proteins and enhances invasion of breast cancer cells^4^. The current investigation determined the expression of FBP17 in invasive ductal carcinomas. In order to assess the correlation between the expression of FBP17 in IDCs with its clinicopathological features, we have investigated FBP17 staining in a series of breast cancer tissues by immunohistochemistry (IHC). In this study, we established that FBP17 express at a higher level in invasive ductal carcinomas as compared to the normal tissues. We also show here the contribution of FBP17 being a proinvasive protein, expressed significantly at a higher level in invasive phenotypes such as HER2 and TNBC. The correlation of expression of FBP17 with lymph node status of tumors as well as histological grade was established. We found that FBP17 is overexpressed in lymph node positive cases of HER2 and TNBC subtypes. Further analysis of FBP17 expression showed its association with high-grade tumors. Taken together, these studies suggest an elevated expression of FBP17 in IDCs determine its poor differentiation and the invasive potential.

## Results

### Clinico-pathological characteristics of tissue microarrays (TMAs)

Patient demographics, tumor grade, expression of receptors, details of age, grade and lymph node status of the cases are summarized in Table 1. All the cases of breast cancer were invasive ductal carcinoma. 8 cases were from normal adjacent tissues. We tested whether FBP17 is expressed in human breast cancer tissues and whether its expression is linked to clinico-pathological features of the breast cancer. The expression of FBP17 was checked using FBP17 specific antibody through IHC. The linkage of FBP17 expression with clinic-pathological features is given in Table 1.

**Table 1:** Patient demographics, tumor grade, expression of receptors, details of age, grade and lymph node status of the patients tissues along with the expression of FBP17 in TMAs. +, ++, +++ in denotes the expression of FBP17 as weak, moderate and strong positive respectively.

Position	Grade	Stage	Type	ER	PR	HER2	Lymph Node Status	FBP17 Expression
A1	3	IIA	Malignant	+	−	0	+	++
A2	2	IIA	Malignant	+	−	3+	+	+
A3	2	IIA	Malignant	−	++	0		0
A4	2	IIA	Malignant	+++	+++	0	−	++
A5	2	IIB	Malignant	*	−	0		0
A6	2	IA	Malignant	+++	+++	2+	−	++
A7	2	IIB	Malignant	++	++	3+		0
A8	2	IIA	Malignant	+++	+++	0		0
A9	2	IIA	Malignant	+++	+	0		0
A10	2	IA	Malignant	+++	+++	0	−	+
B1	2	IIIB	Malignant	+++	+++	0	+	+
B2	2	IA	Malignant	−	−	0		0
B3	2	IIA	Malignant	+++	+++	0	−	+++
B4	2	IIA	Malignant	+++	+	1+	−	++
B5	2	IIIA	Malignant	+++	+++	0	+	++
B6	3	IA	Malignant	*	*	*		0
B7	2	IIA	Malignant	−	−	3+	−	+
B8	2	IIIB	Malignant	+++	+	0	−	++
B9	2	IIA	Malignant	+++	+++	2+	−	+++
B10	2	IIB	Malignant	+++	−	0	+	+++
C1	2	IIA	Malignant	+++	−	0		0
C2	2	IIA	Malignant	+++	++	0	−	+
C3	2	IIA	Malignant	+++	+	0	−	+
C4	2	IIA	Malignant	+++	+++	3+	−	++
C5	2	IIA	Malignant	+++	−	0	−	+++
C6	2	IIB	Malignant	+++	−	3+		0
C7	2	IIA	Malignant	+++	+++	0	−	+
C8	2	IIA	Malignant	+++	+++	2+	−	+++
C9	2	IIA	Malignant	+	−	3+	−	++
C10	2	IIB	Malignant	+++	−	3+	+	+
D1	2	IIB	Malignant	+++	+	0	−	+
D2	3	IIB	Malignant	−	−	0	+	+++
D3	3	IIA	Malignant	+++	+++	0	−	++
D4	–	IIB	Malignant	+++	+	0		0
D5	3	IIB	Malignant	+++	+++	3+	−	++
D6	2	IIA	Malignant	+++	+++	0	−	++
D7	2	IIA	Malignant	−	−	0	−	+
D8	3	IIB	Malignant	−	−	1+	+	+++
D9	2	IIB	Malignant	−	−	3+	−	++
D10	−	IIB	Malignant	+++	+	3+	−	+++
E1	2	IIB	Malignant	+++	+++	0	−	+
E2	3	IIB	Malignant	−	−	3+	−	+++
E3	2	IIIB	Malignant	+++	+++	0	+	++
E4	2	IIIB	Malignant	+++	+	0	+	+++
E5	2	IIB	Malignant	−	−	0	+	++
E6	3	IIB	Malignant	−	−	0	+	+++
E7	3	IIA	Malignant	−	−	0	−	++
E8	2	IIB	Malignant	−	−	3+	+	+++
E9	2	IIB	Malignant	+++	+++	0	+	++
E10	3	IIB	Malignant	−	−	3+	+	+++
F1	2	IIA	Malignant	+++	−	2+	−	+++
F2	2	IIIA	Malignant	−	−	1+	+	+++
F3	2	IIIA	Malignant	−	−	0	+	++
F4	2	IIA	Malignant	+++	+++	0	−	+
F5	2	IIIB	Malignant	+++	+++	0	−	++
F6	2	IIIB	Malignant	−	−	3+	−	+
F7	2	IIIB	Malignant	+++	−	0	+	++
F8	2	IIIB	Malignant	+	−	0	+	+++
F9	2	IIA	Malignant	+++	+++	0	−	++
F10	3	IIIB	Malignant	+++	−	0	+	+++
G1	2	IA	Malignant	++	+	0	−	+
G2	2	IIA	Malignant	−	−	0	−	+++
G3	2	IIB	Malignant	+++	+	0		0
G4	2	IIA	Malignant	−	−	3+	−	++
G5	3	IIA	Malignant	+	−	0	−	+++
G6	3	IIIA	Malignant	−	−	0	+	+++
G7	3	IIIA	Malignant	+	−	3+	+	++
G8	3	IIA	Malignant	+	−	0	−	+++
G9	2	IIA	Malignant	+	−	3+	−	+++
G10	–	IIA	Malignant	−	+	0		0
H1	2	IIA	Malignant	−	−	0		0
H2	3	IIB	Malignant	+++	−	3+	+	+++
H3	3	IIIB	Malignant	+++	+++	0	−	+++
H4	3	IIB	Malignant	+++	+++	0	+	+
H5	3	IIIB	Malignant	−	−	3+	+	+++
H6	3	IIB	Malignant	+++	−	0	+	++
H7	3	IA	Malignant	++	+	3+		0
H8	3	IIA	Malignant	−	−	2+	−	+++
H9	3	IIA	Malignant	−	−	2+	−	+++
H10	3	IIB	Malignant	−	−	0	−	+
I1	3	IIIA	Malignant	−	−	3+	+	+++
I2	3	IIA	Malignant	−	−	0		0
I3	3	IIA	Malignant	+++	−	0	−	+++
I4	3	IIA	Malignant	−	−	3+		0
I5	3	IIA	Malignant	+++	+	0	−	+++
I6	3	IIA	Malignant	−	−	0	−	++
I7	3	IIA	Malignant	+++	+	0	−	+++
I8	3	IIB	Malignant	−	−	2+		0
I9	2	IIB	Malignant	+++	+	0	+	++
I10	3	IIA	Malignant	−	−	0	−	++
J1	3	IIIA	Malignant	+	−	0		0
J2	3	IIA	Malignant	−	−	0	−	++
J3	3	IIA	Malignant	−	−	1+	−	+
J4	2	IIA	Malignant	+++	+	1+	+	++
J5	3	IIB	Malignant	−	−	3+	−	+++
J6	3	IIB	Malignant	−	−	1+	+	+++
J7	3	IIB	Malignant	++	++	0	+	++
J8	3	IIIA	Malignant	−	−	3+	+	+++
J9	3	IIIB	Malignant	−	−	0	+	+++
J10	3	IV	Malignant	++	+	3+	+	++
K1	–	–	NAT	+	+	0		
K2	–	–	AT	+	+	0		
K3	–	–	NAT	+	+	0		
K4	–	–	NAT	+	+	0		
K5	–	–	NAT	+	+	0		
K6	–	–	NAT	*	*	*		
K7	–	–	NAT	+++	++	0		
K8	–	–	NAT	+++	+++	0		
K9	–	–	NAT	+++	+	0		
K10	–	–	NAT	*	*	*		

### FBP17 is markedly upregulated in breast cancer tissues

**Figure 1:**
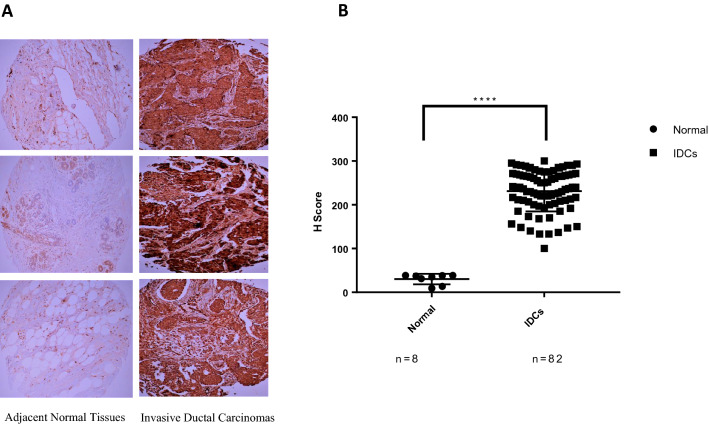
FBP17 expression in IDCs. (**a**) Weak staining of FBP17 in normal adjacent tissues. In IDCs, strong staining of FBP17 was observed (10× magnification). Representative Immunohistochemical staining of FBP17 in IDCs. (**b**) Quantitative estimation of FBP17 expression in normal tissues and IDCs showed a statistically significant difference (*p* < 0.0001).

Our previous observation that FBP17 overexpression in invasive breast cancer cell lines made us inquisitive to determine the levels of FBP17 in breast cancer tissues^4^. To investigate the expression of FBP17 in TMAs, we successfully evaluated the immunohistochemical staining of FBP17 in a total of 90 cases including 8 normal tissues. The tissue microarray core details are given in Table 1 along with the results of the FBP17 protein expression in breast cancer tissues. In normal breast tissues, 6 cases showed absent staining and only 2 cases showed positive staining for FBP17 and both the 2 positive cases displayed weak staining in the ductal epithelia (Fig. 1A). In breast cancer tissues, the staining of FBP17 varied from case to case, majority (42.7%) of the cases fell in the strong expression of FBP17. A significant number (36.6%) of IDCs were found to have moderate to strong immunoreactivity toward FBP17 (Fig. 1B). Representative images from the tissue cores (normal as well as IDCs) expressing FBP17 of varying intensity are shown in Fig. 1A. In order to strengthen the higher expression of FBP17 in breast cancer as observed in IHC, we also subjected the two tumor tissue along with a normal (Imgenex) to immunoblotting. Western blot analysis indicated the elevated levels of FBP17 in tumor tissues (Fig. S1. A). additionally, densitometry analysis of the Western blot demonstrated that expression of FBP17 is increased to 2.6 and 1.7 fold in tumor tissue 1and 2 respectively (Fig. S1. B). The analysis of FBP17 levels in breast cancer tissues by IHC and Western blot suggest the significant elevation of FBP17. For IHC we used FBP17 specific antibody that we have validated in our previous study in immunoblotting and immunoprecipitation^4^. For the present study, we validated the antibody by immunofluorescence on FBP17-Knock down cells (Fig. S2).

### Profiling of FBP17 expression in IDCs correlates with invasive molecular subtype

**Figure 2:**
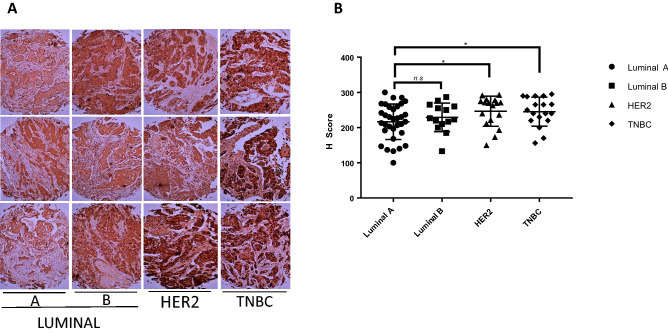
Elevated levels of FBP17 in invasive molecular subtypes of breast cancer. (**a**) Weak staining in Luminal A, whereas moderate to strong staining can be depicted in Luminal B, HER2 and TNBC subtype. Representative immunohistochemical staining of FBP17 among molecular subtypes. (**b**) Quantitative analysis of FBP17 staining among different molecular subtypes showed statistical difference in invasive phenotypes such as HER2 and TNBC (*p* < 0.05).

Invasive ductal carcinomas are heterogeneous in nature and express different receptors and thus categorized into different molecular subtype^17,18^. The TMAs that we have employed in this study, were with known ER, PR and HER2 status. To determine the levels of FBP17 in these molecular subtypes, we assigned the IDCs in the TMAs into Luminal A, Luminal B, HER2 and TNBC subtypes. Luminal A tumors (ER and/or PR) comprised of the majority, 33 cases followed by Luminal B tumors (ER/PR/HER2) that represented 14 cases. HER2 (only HER2 expression) represented 17 cases and TNBC (lack of three receptors) 18 cases. Then we extended the analysis of FBP17 staining to the major molecular subtypes. The image analysis of the tissue cores was done by image J software as well as manually. We found the moderate to strong FBP17 expression was present in all of the molecular subtypes. Pathological analysis of the IHC staining in these subtypes revealed the higher expression of FBP17 in TNBC and HER2 (Fig. 2). The tissue cores from the HER2 and TNBC subtypes showed a stronger staining as compare to the Luminal subtype (Fig. 2A). A significant observation was made for increased FBP17 expression levels in HER2 and TNBC subtypes as compared to the luminal subtypes (p ≤ 0.05) (Fig. 2B). Cancers belonged to Luminal A had a moderate staining and interestingly, luminal B cancers were also found to express higher levels of FBP17 (Fig. 2A, B). The expression of FBP17 in different molecular subtypes was further investigated for quantitative analysis (Fig. 2B). Having established FBP17 expression in invasive ductal carcinomas (Fig. 2A, B), we expanded the analysis of FBP17 expression in terms of invasiveness of the cancers. As expected in terms of invasiveness, HER2 and TNBC cancers being the highly invasive cancers significantly showed higher number of cases with strong immunostaining of FBP17. HER2 and TNBC cases showed the higher expression in 58.8% and 50% respectively while luminal A displayed higher FBP17 expression in less than 30% cases. Luminal B showed 42.9% of cases with high levels of FBP17 (Fig. 2B). The analysis of FBP17 immunostaining in these IDCS indicate that FBP17 elevated expression (Table2) is elevated in breast cancers lacking the expression of ER.

**Table 2:** Correlation of FBP17 expression with clinic-pathological characteristics of IDCs.

Characteristics	Total No. cases (N = 82)	FBP17 IHC staining intensity					
		FBP17 low (n = 17)		FBP17 moderate (n = 30)		FBP17 high (n = 35)	
		FBP17 low	Low %	FBP17 moderate	Moderate %	FBP17 high	High %
**Tumor grade**							
Grade 2	43	13	30.2	18	41.8	12	28
Grade 3	39	4	10.2	12	30.8	23	59
**Lymph node status**							
LN+	34	4	11.8	13	38.2	17	50
LN−	48	13	27.1	17	35.4	18	37.5
**Molecular subtypes**							
Luminal A	33	10	30.3	13	39.4	10	30.3
Luminal B	14	2	14.2	6	42.9	6	42.9
HER2	17	3	17.6	4	23.6	10	58.8
TNBC	18	2	11.1	7	38.9	9	50

### High expression of FBP17 is associated with the lymph node positive tumors of invasive subtypes

**Figure 3:**
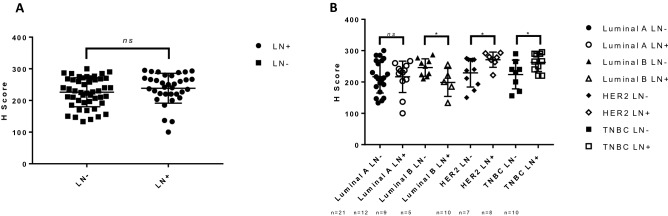
Association of FBP17 expression and lymph node status. (**a**) Quantitative analysis the immunostaining by imaging software revealed no statistically significant difference in FBP17 levels in case of lymph node positive and negative tumors (*p* > 0.05). (b**)** Quantification of FBP17 expression in the lymph node positive and negative cases among different molecular subtypes (*p* < 0.05).

Axillary lymph node (LN) metastases remain the most important factor for breast cancer diagnosis and prognosis. Subsequently, we investigated the association of FBP17 with clinicopathological features. Therefore, we determined the FBP17 levels in lymph node positive and negative cancers. The total numbers of lymph node negative and positive cases were 48 and 34 respectively. Both node negative and positive tumors did not show any significant differences in the intensity of the staining (Fig. 3A). This result came as a surprise, given the role of FBP17 in invasion^4,16^. Further dissection of FBP17 expression in lymph node positive case of invasive phenotypes (Table 2), we expanded the analysis of FBP17 in the node negative and positive tumors in terms of molecular subtypes. We dissected the IDCs into different molecular subtypes in terms of node negative and positive cases. Interestingly, we found no significant difference in the levels of FBP17 in node negative and positive tumors in Luminal A subtype (Fig. 3B). Rather LN (Lymph node) positive cases in this subtype showed lower levels of FBP17 than LN negative cases. However, node negative and positive tumors in Luminal B subtype displayed a significant difference in the expression of FBP17 (*p* ≤ 0.05) (Fig. 3B). As expected, in terms of invasive subtypes, both HER2 and TNBC showed the expression of FBP17 significantly higher in lymph node positive tumors (*p* ≤ 0.05) (Fig. 3B). Results from these studies indicate the statistically significant association of FBP17 expression with the invasive ability of the cancer cells.

### High FBP17 expression positively correlates with histological tumor grade

**Figure 4:**
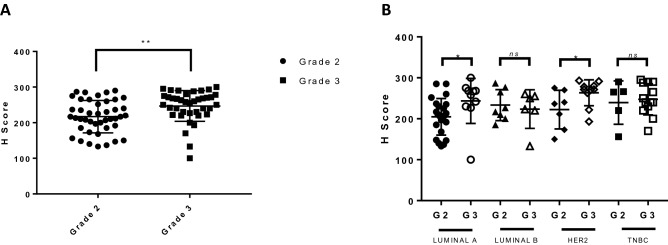
Correlation between FBP17 expression and grades of IDCs. (**a**) Statistical significant difference in FBP17 expression in grade 3 tumors as compared to grade 2 (*p* < 0.05). (b**)** Quantitative analysis showed statistically significant differences between grade 2 and grade 3 tumors among the Luminal A and HER2 molecular subtypes (*p* < 0.05).

Breast cancer is a heterogeneous disease and the cells in the same tumor display different levels of differentiation. The measurement of mitotic activity and differentiation predicts tumor grade and it is a well-accepted prognostic marker in early stage of breast cancer^19,20^. The differentiation of the tumors is an important diagnostic and prognostic factor^21^. The tissue microarrays we have used in the study comes with a grading of the tumor. No tumor was of grade 1, so all the cases were classified either into grade 2 or grade 3. In order to determine if any significant FBP17 expression difference in grade 2 and grade 3 tumors, we expanded the analysis of IHC and found that grade 3 tumors significantly demonstrated higher expression of FBP17 (*p* ≤ 0.01) (Fig. 4A) indicating the role of FBP17 in the poor differentiation of tumors. The analysis of IHC in terms of tumor grade was further dissected in context of molecular subtypes. Surprisingly, the expression of FBP17 was found to be significantly high in poorly differentiated tumors in case of Luminal A molecular subtype. The similar trend was observed for HER2 subtype. However, no significant differences in the expression of FBP17 were observed in grade 2 and grade 3 tumors in Luminal B and TNBC subtypes (Fig. 4B). These studies are interesting and surprising that FBP17 can play a role in the differentiation of the tumors.

### Expression of FBP17 correlates with the low survival rate

**Figure 5:**
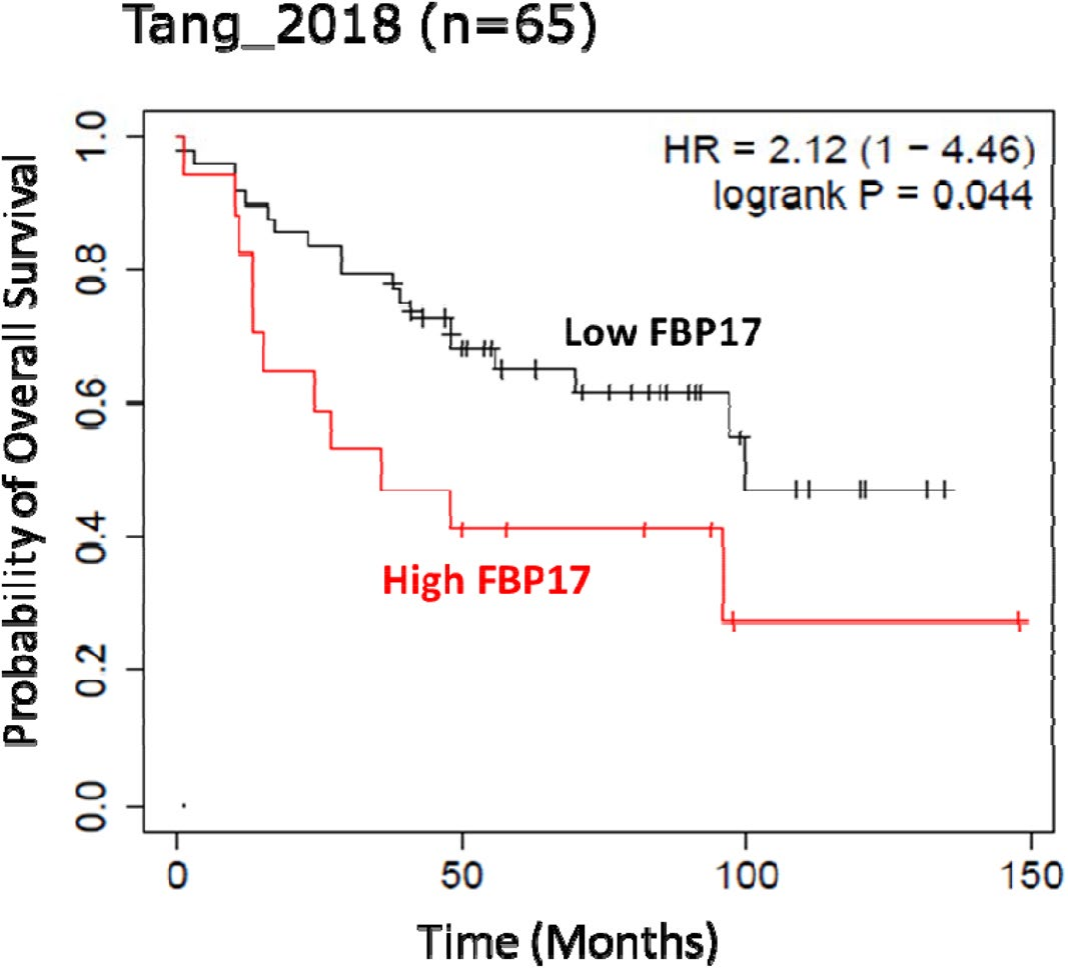
Kaplan–Meier Plotter analysis of basal breast cancer patients with high and low FBP17 transcript levels according to overall survival (n = 65; HR = 2.12, logrank* p* = 0.044).

In order to correlate the expression of FBP17 in breast cancer tissues to the patient outcome, we have analyzed publicly available dataset, Tang_2018, Kaplan-Meier Plotter^22^ for *FNBP1* (gene symbol for FBP17) and Q96RU3 (Uniprot ID for FBP17). The analysis suggested that the higher expression of FBP17 protein in breast cancer patients is significantly associated with the low survival (*p* = 0.04) (Fig. 5). Thus our results indicate that increased levels of FBP17 in breast carcinomas have pathological implications and might be associated with outcome of the disease.

## Discussion

Breast cancers that are of invasive phenotype have activated signaling pathways leading to metastasis^23,24^. Activation of EGFR/HER2 and Src signaling pathways lead to the formation of structures such as invadopodia augments invasion and metastasis^25-28^. We previously reported that FBP17 is overexpressed in invasive breast cancer cell lines promotes invadopodia formation and invasion of cancer cells^4^. We were interested to explore the expression of FBP17 in breast cancer patients. To investigate, we subjected the invasive ductal carcinoma tissue microarrays to immunohistochemistry (IHC) staining for FBP17. The IHC intensity score indicates higher FBP17 expression in IDCs when compared with the normal adjacent tissues. Further, IHC analysis of IDCs revealed that the concentrated staining in the cytoplasm was consistent with the localization of FBP17. In normal adjacent tissues, FBP17 staining was much weaker when compared to the IDC sections, where we found moderate to strong staining. However, there were few IDCs that displayed weaker staining.

The observation that a large number of IDCs showing higher expression of FBP17 associates with its expression in invasive breast cancer cell lines and function in the invasion of cancer cells^4,16^. Among the molecular subtypes of breast cancers, HER2 and TNBC are considered to be aggressive and highly invasive^17^. These cancers are characterized by a high rate of malignancy, recurrence, metastasis and poor prognosis^18,29^. In the current study, we found that lymph node positive tumors didn’t show a significant difference in the FBP17 expression from the lymph node negative tumors. This observation was surprising, given the contribution of FBP17 in invasion of cancer cells. We then investigated and assessed the data in terms of different subtypes of cancer. Data suggested an elevation of FBP17 intensity score in lymph node positive tumors of Luminal B, HER2 and TNBC when compared to Luminal A subtype. The observation suggests the heterogeneity of the disease and emphasizes the need to discover distinguished markers for various subtypes. The expression of FBP17 might be raising the threshold of invasiveness of cancer cells. Notably, here we report the elevated levels of FBP17 in the HER2 and TNBC subtypes, which supports the hypothesis of the upregulation of FBP17 in promoting the process of invasion.

Breast cancer is a multifactorial and heterogeneous disease. The tumor differentiation varies from histological grade 1 to grade 4. Heterogeneous gene expression among different histological grade tumors has been established^30^. In our TMAs, we found that IDCs of higher histological grade had a higher levels of FBP17 as compared to grade 2. This analysis exposed the relationship between the FBP17 and differentiation of the tumors. The significant difference in the expression of FBP17 between grade 2 and grade 3 made us questioning to explore further that if there is a relationship between grade 2 and grade 3 tumors of different molecular subtypes. It was interesting to note that grade 2 and grade 3 tumors had no significant differences in FBP17 expression in Luminal B and TNBC subtype. However, the expression of FBP17 was significantly higher in Luminal A and HER2 subtypes. This observation becomes more important in relation to the heterogeneous diseases like breast cancer of various subtypes harboring mutations in several tumor suppressor genes. The scientific literature completely lacks the information on the role of FBP17 in tumor differentiation. We are the first to designate this relationship and it would be worth to explore and elucidate the relationship further to demonstrate whether FBP17 has any role to play in cell cycle progression, proliferation and differentiation of tumors. The role of FBP17 in the differentiation of tumors among various molecular subtypes warrant further studies. The histological grade is based on mitotic index, polymorphism and differentiation^21^. The observation of FBP17 expression among various subtypes in context of histological grading raises the need to study the tumors based on their clinical and biological factors associated with the inherent aggressiveness of the disease. The sub-classification of the molecular subtypes based on the expression and function of molecular markers might contribute to the efficient therapeutic regimen for the disease. The increased risk of low survival of breast cancer patients would fit with our evidence for the higher expression of FBP17 in invasive and poorly differentiated breast tumors.

To conclude, this is the first study to demonstrate the expression of FBP17 in invasive ductal carcinomas. We showed that FBP17 was markedly overexpressed in breast cancer tissues. The levels of FBP17 were elevated in molecular subtypes that are characterized with higher rates of invasion. The current study also implicates high expression of FBP17 in lymph node positive tumors of Luminal B, HER2 and TNBC subtypes as compared to Luminal A. The study indicated that FBP17 is significantly elevated in poorly differentiated tumors. The data presented here raises the possibility of the roles that FBP17 plays are far broader than assumed. Future studies are considerably essential to sub classify the molecular subtypes for clinical management of the breast cancer.

## Methods

### TMAs and antibodies

IHC of TMAs was carried out as described previously^5,8^. Human breast cancer tissue microarrays (TMAs) (BC081120c) were purchased from US Biomax, Inc., Rockville, MD, USA consisting 110 cores, from which 100 were of breast cancer cases and 10 cores were of normal tissue cores. The TMAs were provided with the data on the basis of the expression of receptors (ER, PR and HER2), lymph node status, clinical and grade of the tumors. Out of 100 Breast cancer tissue cores, 82 were selected and from 10 normal cases, only 8 cases were selected for further analysis of the study. Cores that showed poor staining of less than 10% cells are not included in the study. Rabbit Anti- FBP17 antibody was obtained from Novus Biologicals (Cat. No: NBP1-47260).

### Immunohistochemistry (IHC) detection, analysis and scoring of FBP17 staining

TMAs were stained using the Discovery XT Staining System (Ventana Medical Systems, Inc. Tucson, AZ, USA). IHC of the TMAs were done at the Department of Histopathology, PGIMER, Chandigarh. Antigens were retrieved with an EDTA pH 8.0 solution and incubated with FBP17 antibody at dilution 1:100. Staining was visualized with DAB treatment and a Hematoxylin counterstain. Analysis of the microarrays was done and Histological scoring (H Score) was given according to the percentage of cells stained (0 is <10%, 1+ is 10–25%, 2+ 25–50%, 3+ is 50–75%, 4+ above 75%) and intensity. The staining intensity of FBP17 was scored as weak, moderate and strong positive according to the brown intensity at the cytoplasmic portion. Similarly, we also analyzed the same tissue microarrays in terms of other parameters such as molecular subtypes, lymph node and grade of the tumor.

### Statistical analysis

Statistical analysis was done using the GraphPad Prism software (GraphPad Prism version 7.00 for Windows, GraphPad Software, La Jolla California USA, www.graphpad.com) and Excel 2013 (Microsoft). An independent pathologist and a researcher gave scoring for the obtained data. Data was summarized and the statistical significance of the data of various groups with same parameters was incorporated and analyzed using unpaired *t*-test for 2-group comparison. *P* value < 0.05 was considered as statistically significant.

## Supplementary information

Supplementary Legends.

Supplementary Figures S1 and S2.

## Abbreviations

CIP4: CDC42 interacting protein 4
FBP17: Formin binding protein 17
ECM: Extracellular matrix
EGFR: Epidermal growth factor receptor
ER: Estrogen Receptor
PR: Progesterone receptor
F-BAR: Fer/cip-bin amphyphysin RVS
HER2: Human epidermal growth factor receptor 2
IDCs: Invasive ductal carcinomas
IHC: Immunohistochemistry
WASP: Wiskot Aldrich syndrome protein
TMAs: Tissue microarrays
TNBC: Triple negative breast cancer
Toca-1: Transducer of Cdc42-dependent actin assembly protein 1
LN: Lymph node
AN: Adjacent normal
