# Supplementary material for: High formin binding protein 17 (FBP17) expression indicates poor differentiation and invasiveness of ductal carcinomas
Source: Sci Rep. 2020 Jul 14;10:11543. doi: 10.1038/s41598-020-68454-9 (PMC7360568; doi:10.1038/s41598-020-68454-9)
Supplement: Supplementary file 1 — Supplementary Legends. [file 41598_2020_68454_MOESM1_ESM.pdf]

Fig. S1.A. Higher Expression of FBP17 in tumor lysates. FBP17 antibody was used for the detection in tumor lysate and GAPDH antibody was used as a loading control.

Fig. S1.B. Graph depicts the higher expression of FBP17 as measured by densitometry in tumor lysates.

Fig. S2. Reduced expression of FBP17 in FBP17-KD-MDAMB-231 cells. Instance staining in MDAMB 231 cells and reduced staining in MDAMB-KD cells indicates the specificity of FBP17 antibody. Normal IgG was used as a control.
