# Supplementary figures and images for: High formin binding protein 17 (FBP17) expression indicates poor differentiation and invasiveness of ductal carcinomas
Source: Sci Rep. 2020 Jul 14;10:11543. doi: 10.1038/s41598-020-68454-9 (PMC7360568; doi:10.1038/s41598-020-68454-9)

Fig.S1.A

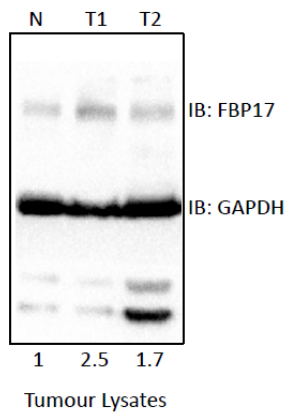

Fig.S1.B

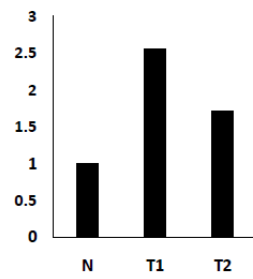

Fig.S2

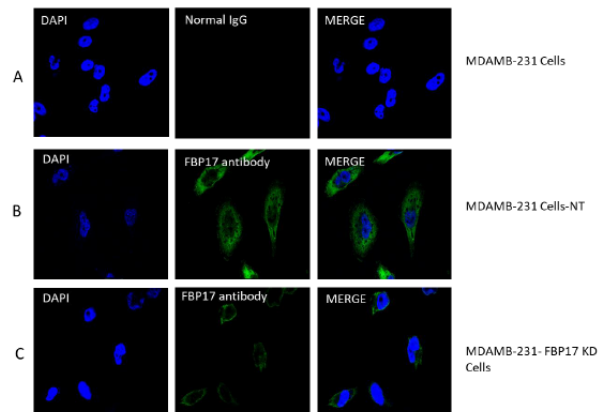

Supplement: Supplementary file 2 — Supplementary Figures S1 and S2. [file 41598_2020_68454_MOESM2_ESM.pdf]
